# Exposure to NO_2_, CO, and PM_2.5_ is linked to regional DNA methylation differences in asthma

**DOI:** 10.1186/s13148-017-0433-4

**Published:** 2018-01-05

**Authors:** Mary Prunicki, Laurel Stell, Deendayal Dinakarpandian, Mariangels de Planell-Saguer, Richard W. Lucas, S. Katharine Hammond, John R. Balmes, Xiaoying Zhou, Tara Paglino, Chiara Sabatti, Rachel L. Miller, Kari C. Nadeau

**Affiliations:** 1 Sean N. Parker Center for Allergy and Asthma Research at Stanford University, Stanford, CA 94305 USA; 2 Department of Medicine, Stanford University, Stanford, CA 94305 USA; 3 Department of Biomedical Data Science, Stanford University, Stanford, CA 94305 USA; 4 Center for Biomedical Informatics Research, Stanford University, Stanford, CA 94305 USA; 5 Department of Medicine, Columbia University, New York, NY 10032 USA; 6 Department of Statistics, Stanford University, Stanford, CA 94305 USA; 7 Southwest Environmental Institute, Phoenix, AZ 85087 USA; 8 School of Public Health, University of California, Berkeley, Berkeley, CA 94720 USA; 9 Department of Medicine, University of California, San Francisco, San Francisco, CA 94143 USA; 10 Division of Pulmonary and Critical Care Medicine, Department of Medicine, Stanford University, Stanford University School of Medicine, 269 Campus Drive, CCSR 3215, MC 5366, Stanford, CA 94305-5101 USA

**Keywords:** Ambient air pollution, Immune system, Regulatory T cell, Epigenetics

## Abstract

**Background:**

DNA methylation of CpG sites on genetic loci has been linked to increased risk of asthma in children exposed to elevated ambient air pollutants (AAPs). Further identification of specific CpG sites and the pollutants that are associated with methylation of these CpG sites in immune cells could impact our understanding of asthma pathophysiology. In this study, we sought to identify some CpG sites in specific genes that could be associated with asthma regulation (*Foxp3* and *IL10*) and to identify the different AAPs for which exposure prior to the blood draw is linked to methylation levels at these sites. We recruited subjects from Fresno, California, an area known for high levels of AAPs. Blood samples and responses to questionnaires were obtained (*n* = 188), and in a subset of subjects (*n* = 33), repeat samples were collected 2 years later. Average measures of AAPs were obtained for 1, 15, 30, 90, 180, and 365 days prior to each blood draw to estimate the short-term vs. long-term effects of the AAP exposures.

**Results:**

Asthma was significantly associated with higher differentially methylated regions (DMRs) of the *Foxp3* promoter region (*p* = 0.030) and the *IL10* intronic region (*p* = 0.026). Additionally, at the 90-day time period (90 days prior to the blood draw), *Foxp3* methylation was positively associated with NO_2_, CO, and PM_2.5_ exposures (*p* = 0.001, *p* = 0.001, and *p* = 0.012, respectively). In the subset of subjects retested 2 years later (*n* = 33), a positive association between AAP exposure and methylation was sustained. There was also a negative correlation between the average *Foxp3* methylation of the promoter region and activated Treg levels (*p* = 0.039) and a positive correlation between the average *IL10* methylation of region 3 of intron 4 and *IL10* cytokine expression (*p* = 0.030).

**Conclusions:**

Short-term and long-term exposures to high levels of CO, NO_2_, and PM_2.5_ were associated with alterations in differentially methylated regions of *Foxp3*. *IL10* methylation showed a similar trend. For any given individual, these changes tend to be sustained over time. In addition, asthma was associated with higher differentially methylated regions of *Foxp3* and *IL10*.

**Electronic supplementary material:**

The online version of this article (10.1186/s13148-017-0433-4) contains supplementary material, which is available to authorized users.

## Background

Asthma is the most frequent chronic disease in children [1], and its prevalence continues to increase, raising global public health concerns [2–4]. Previous studies found that high prenatal ambient air pollutant (AAP) exposure alters epigenetic programming in utero [5] and that children are at greater risk of developing asthma when exposed to higher concentrations of AAPs [6]. This further raises concern and increases the urgency of understanding the relationship between AAP exposures and asthma and finding effective ways to prevent, treat, and/or cure asthma. Although the mechanisms by which exposures to AAPs increase asthma prevalence remain poorly understood, evidence suggests that AAPs can mediate T cell polarization and cytokine dysregulation via epigenetic modifications [7–10].

DNA methylation at least partially controls the expression of certain key genes that are known to be involved in immune tolerance [11–13]. We previously demonstrated that exposure in children to high levels of polycyclic aromatic hydrocarbons (PAHs), a component of AAPs, is associated with higher overall percent methylation of the transcriptional regulatory region of the forkhead box transcription factor 3 (*Foxp3*) locus [8]. In a subsequent study, in addition to the presence of higher *Foxp3* methylation levels, we found that exposure to higher levels of PAHs is associated with decreased expression of *IL10*, an anti-inflammatory cytokine. These associations were stronger in children with asthma, suggesting disease-linked effects [14]. Other studies have also associated air pollution with other immune parameters, such as the association of exposure to NOx with decreases in *IL10* cytokine expression and exposure to diesel exhaust with higher differentially methylated regions (DMRs) of *Foxp3* [15]. A recent review of ambient air pollutants (AAPs), epigenetic regulation, and asthma highlights the challenge that asthma is heterogeneous, responsiveness to traffic-related air pollution, and may be a distinct phenotype [16]. In addition, a study by Clifford et al. [17] found DNA methylation changes after diesel exhaust exposure in more than 500 CpG sites in the bronchial epithelial cells. Finally, AAPs and DNA methylation have been linked to other health problems, such as cardiovascular disease [18].

We sought to identify CpG sites in the *Foxp3* and *IL10* genes that have higher DMRs in children with asthma exposed to AAPs. Our primary hypothesis was that exposure to high levels of CO, O_3_, NO_2_, and PM_2.5_ would be associated with higher DMRs within the *Foxp3* gene and the intron 4 region of the *IL10* gene. The Foxp3 gene regulates transcription of Tregs, and the Il10 gene regulates transcription of various cell types, including CD4^+^ T cell populations, because it has been shown to act as an enhancer element [19]. In addition, our secondary hypotheses were that methylation would be sustained following continued exposure to these pollutants and that Treg levels would be negatively associated with *Foxp3* methylation at specific CpG loci in the enhancer and promoter regions.

## Methods

### Subjects

We recruited subjects from Fresno, California, where there is consistently high AAP. According to the 2017 American Lung Association “State of the Air” report, Fresno ranked third in the country for year-round and short-term particle pollution and O_3_ exposure [20]. Fresno County also has a high childhood asthma prevalence in comparison to other counties in California (24 vs. 17%, ages 5–17) [21]. Recruiting from the Fresno Unified School district from 2010 to 2015 resulted in a convenience sample of 198 children with complete data, 188 of which were included in the analysis after quality control, with a median age of 14.7 years. In addition, we retested 33 subjects after approximately 2 years (mean 820 days, SD 243 days; again, as a convenience sample).

At each clinical visit, information on BMI, a detailed health and general history questionnaire, and blood samples were obtained. Subjects were excluded if they had taken oral immunosuppressants within 5 days of the blood draw, had a history of allergen immunotherapy within 1 year of the clinical visit, had a chronic disease other than allergies or asthma, or had an acute infection. In addition, no blood specimen was taken during an infection, sickness, or during the use of oral steroids. Subjects with asthma were defined by the participant’s report of a physician’s diagnosis of current asthma. Secondhand smoke exposure (SHS) was defined as exposure to cigarette smoke from other household members. Participant smoking was defined as smoking more than 100 cigarettes in their lifetime.

### Collection and processing of blood specimens

Human peripheral blood mononuclear cells (PBMCs) and plasma were extracted from the blood via Ficoll procedure and stored in liquid nitrogen and at − 80 °C as per published techniques [22].

### Methylation analysis

We first selected CpG sites within the upstream promoter (− 138, − 126, − 77, − 65, − 58, and − 15) and enhancer (− 4506, − 4500, − 4494, and − 4484) regions of *Foxp3* (on the X chromosome) located on the human genome (hg38) chrX at nucleotides 49264838, 49264826, 49264777, 49264765, 49264758, 49264715, and 49269206, 49,69200, 49269194, 49269184, respectively, and within the intron 4 region of *IL10* (+ 2888, + 2907, + 2921, + 3261, + 3265, and + 3281 on chromosome 1), located on the human genome (hg38) chr1 at nucleotides 206769607, 206769588, 206769574, 206769234, 206769230, 206769214, respectively, as these sites have been shown in the literature to be key in modulating regulatory T cell (Treg) response and overall immune tolerance [11, 19, 23]. We then examined associations between exposures to CO, O_3_, NO_2_, and PM_2.5_ and methylation levels at these CpG sites. To determine whether methylation of CpG sites is maintained with long-term exposure, we repeated measurements on a subset of samples obtained at approximately 2 years after the initial blood samples were obtained. Methylation analysis using the pyrosequencing approach was performed on the following gene loci: ten CpG sites for *Foxp3* and six CpG sites for *IL10*. Bisulfite modification and subsequent DNA amplification of specific CpG regions within the *Foxp3* and *IL10* genes were conducted by PCR using the different pairs of primers for each region enumerated in Additional file 1: Table S1. Please see Additional file 1 for further details.

### CyTOF mass cytometry

We also examined Treg, *Foxp3*, and *IL10* protein expression in a subset of the subjects using mass cytometry (CyTOF). CyTOF uses antibodies tagged with heavy metal ions and allows for identifying many more cell markers in a single sample than flow cytometry and without issues of spectral overlap. Here, we performed CyTOF on a randomly selected subset of subjects (*n* = 74; 24.3% asthmatics, 75.7% non-asthmatics) for which additional blood was available from the same blood draw as that used for methylation analysis to assess for cell composition differences. Briefly, PBMCs were thawed, stimulated for 4 h with 20 ng/mL phorbol myristate acetate + 1 μg/mL ionomycin (PMA-ION), and stained for surface markers, intracellular markers, and transcription factors using CyTOF. Please see Additional file 1 for further details.

### Cell composition analysis

Nine cell subtypes were measured by gating on cell surface markers obtained from the CyTOF analysis: B Cells, monocytes, CD3+ cells, CD4+, CD8+, Th17 cells, Tregs, Th1, and Th2 cells. The cell counts were converted into non-overlapping proportions, with an additional miscellaneous category (the remaining fraction of unmeasured cell types, typically < 5%) accounted for to normalize the distribution to 100%. The resulting cell distributions were compared between the asthmatic and healthy groups and used as covariates to confirm the reproducibility of differences in methylation (see Additional file 1: Table S2).

### Ambient air pollution exposure estimation

Exposure to AAPs was estimated using hourly concentration data [24] of four common pollutants (CO, O_3_, NO_2_, and PM_2.5_) measured at four air quality monitoring stations located within Fresno city limits. These data were acquired from the US Environmental Protection Agency’s Air Quality System online database (https://www.epa.gov/aqs).

In order to capture both the spatial and temporal variation in the AAP exposure of each participant, we computed an index of the average outdoor residential AAP exposure for each individual using inverse distance weighting (IDW) [25, 26] of AAP measurements from the air quality monitoring stations according to their distance from each participant’s residence. For each blood sample and air pollutant, exposure was defined as the mean of the IDW values over the given time period (1, 15, 30, 90, 180, or 365 days) before the sample was taken. See online Additional file 1 for further details of these data processing choices.

### Statistical analysis

Statistical analysis was performed using the R statistical computing software (R 3.4.1). We used linear regression to adjust methylation values for sex, age, and BMI. Using multivariate linear regression, we evaluated associations of resulting residuals with asthma status and ambient air pollution.

Due to the presence of related subjects within our study, we also considered linear mixed-effects models fit using generalized least squares, as implemented in the “gls” function in the “nlme” R package (version 3.1.131) for non-linear mixed-effects models. Unless explicitly stated otherwise, the results from fixed-effects models are described in the main text, and the results from mixed-effects models are described in Additional file 1. Detailed descriptions of all analyses are available in Additional file 1, along with de-identified data, exposure data, and R code (see Additional files 2, 3, 4, and 5).

Multivariate linear regression was repeated on a random subset of the data for which CyTOF-derived cell counts were obtained. The proportions of different cell types were incorporated into the model to adjust for any differences. Additionally, we performed *t* tests for each cell type to look for statistically significant differences in cell distribution. Family-wise multiple testing correction was performed by using the Benjamini-Hochberg procedure [27] with a false discovery rate of 0.05.

## Results

### Subject characteristics

For the 188 children (50% female, 50% male) included in the study analysis, the median age (quartile 1; quartile 3) was 14.68 years (12.98; 16.87). Median body mass index (BMI) (quartile 1; quartile 3) was 21.81 kg/m^2^ (18.97; 26.28). No significant differences in these variables (*p* > 0.05) were seen between the two groups (35.6% asthmatics vs. 64.4% healthy individuals, Table 1).

**Table 1:** Demographic characteristics of study subjects*

	Non-asthmatic	Asthmatic
Number of subjects	121	67
Total number of male	59 (48.8%)	35 (52.2%)
Median age at enrollment (years) (quartile 1; quartile 3)	14.7 (12.9; 16.9)	14.6 (13.1; 16.9)
Median BMI (kg/m^2^) (quartile 1; quartile 3)	21.7 (19.1; 25.6)	22.0 (18.8; 28.0)
Total number of rhinitis	9 (7.8%)	12 (23.1%)
Total number of eczema	11 (9.8%)	8 (15.7%)
Total number of African-American	4 (3.0%21)	5 (7.5%)
Total number of Hispanic or Latino	72 (59.5%)	27 (40.3%)
Total number of Caucasian	34 (28.0%)	15 (22.4%)
Total number of mixed race	5 (4.1%)	3 (4.5%)
Total number of not reported	6 (5.0%)	17 (25.3%)
Total number of exposed to secondhand smoke	12 (10.3%)	7 (12.5%)
Total number of smokers	1 (0.9%)	0 (0%)
Total number of household income < $15,000	35 (28.9%)	15 (22.4%)
Total number of household oncome $15,000–30,000	27 (22.3%)	12 (17.9%)
Total number of household income $31,000–50,000	12 (9.9%)	3 (4.4%)
Total number of household income > $50,000	35 (28.9%)	18 (26.9%)
Total number of household income not reported	12 (9.9%)	19 (28.3%)

*BMI* body mass index

^*^Frequencies may not sum to full sample size due to missing data

### Air pollution

**Fig. 1:**
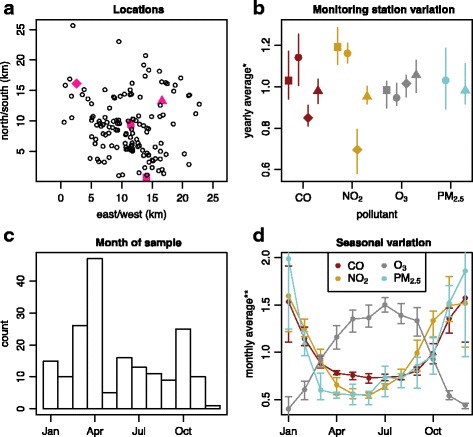
Panel **a** shows the relative locations of the air quality monitoring stations (magenta symbols) and the participant home addresses (black circles). Panel **b** illustrates the variability across monitoring stations (indicated by different symbols as in the legend), which match the shapes of the magenta symbols in panel **a** for the four studied pollutants (CO, NO_2_, O_3_, PM_2.5_), indicated with different colors as in the legend in panel **d**, using yearly averages across the study period (2010–2015); the bars show the ranges of the annual averages. Only two monitoring stations reported values for PM_2.5_. Panel **c** describes the distribution of the 188 clinic visits across months of the calendar year, and panel **d** illustrates the seasonal variation in pollutants using monthly averages across monitoring stations, with the bars showing the ranges of the monthly averages across years 2010–2015. Note: In panels **b** and **d**, each value is divided by the average for the pollutant overall monitoring stations and years. Also, two stations in close proximity replaced each other, so their data are combined in panels **b** and **d**; in panel **a**, they are indicated by two magenta circles very close together in the middle of the plot

Exposures to pollution were averaged over 1, 15, 30, 90, 180, and 365 days prior to each convenience sampling blood draw. All subjects lived within 10 km of at least one of the monitoring stations (Fig. 1a). Annual means of data recorded at each monitoring station showed limited differences between stations (Fig. 1b). Clinic visitations by subjects varied with season with the maximum number of visits occurring in April (Fig. 1c). Substantial seasonal variations were observed in the concentrations of the air pollutants over a 12-month period (Fig. 1d). A more detailed comparison of the pollution levels between air quality monitoring stations and years in Additional file 1: Figure S1 shows that there is more variability between stations than there is between yearly averages from a single station. The results of inverse weighted averaging of the pollutants on January 1, 2014, over a rectangular grid covering all participant locations are shown in Additional file 1: Figure S2, revealing that the estimate is smooth with a local extremum at each monitoring station. The distribution of pollution exposure across individuals for six different durations prior to the visit is shown in Additional file 1: Figure S3. Because subjects did not differ substantially in their average AAP exposure over the course of a year (as indicated by very short boxes for this time period in Additional file 1: Figure S3), we excluded the 365-day time period from further analyses. We defined short-term as time periods equal to or less than 90-day exposure. Long-term was defined as greater than 90-day exposure.

### Normalization of methylation data

**Fig. 2:**
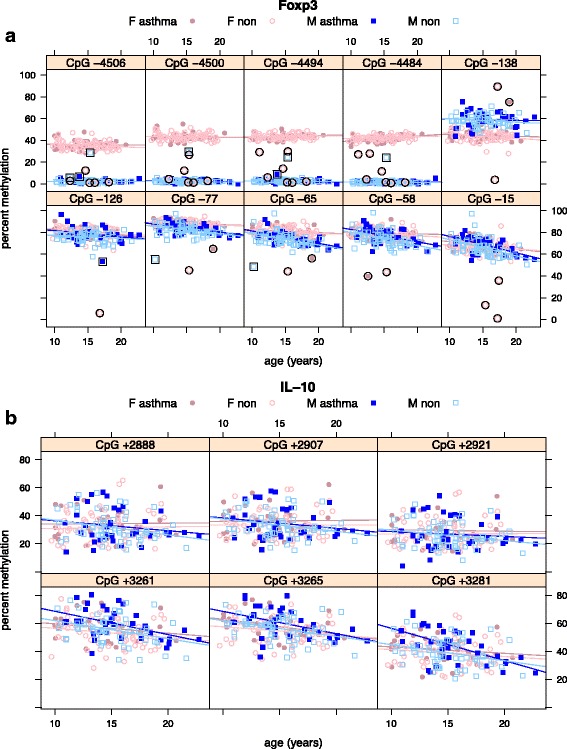
Foxp3 (**a**) and IL-10 (**b**) percent methylation at measured CpG sites as a function of age, grouped by sex (M/F), and asthma status (non/asthma) as indicated. Markers of Foxp3 outliers are enclosed by black circles or squares, depending on sex. These outliers are not used in the computation of the regression lines shown for each group

**Fig. 3:**
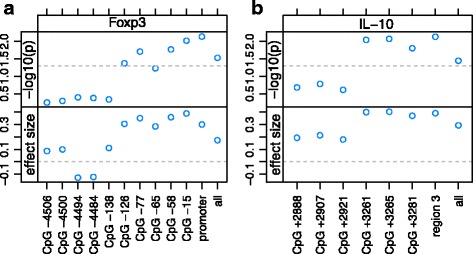
The *p* values and effect sizes from ANOVA tests of significance of asthma status on normalized methylation for the Foxp3 (**a**) and IL-10 (**b**) genes at individual sites, and also averages over groups of sites. The horizontal dashed lines in the *p* value plots indicate *p* = 0.05. In the plots of effect sizes, the dashed lines indicate *y* = 0

Before testing our primary hypothesis, we normalized the effects of sex, age, and BMI on *Foxp3* and *IL10* methylation. Figure 2 shows the relationship between these methylation values and sex and age from 241 visits by 198 eligible subjects, revealing a few outlier values of *Foxp3* percent methylation. Outliers were defined as values farther than three times the interquartile range (IQR) from the first quartile or third quartile; these values were excluded from the analysis presented in the main text, leaving *n* = 188 individuals. In the supplement, we report the results we would have obtained if we had not excluded these outliers, showing that the main conclusions would not have changed substantially. Figure 2a also shows that *Foxp3* DMRs in females were greater than in males and that, at the promoter sites, it decreased with age (*n* = 188, *p* < 0.0001; Additional file 1: Table S3). The relationship with sex is consistent with the presence of a hypermethylated, inactive X chromosome. The only exception was CpG-138, which had a higher DMR in males than in females (Additional file 1: Figure S4). BMI may be weakly associated with *Foxp3* methylation when adjusted for sex, age, and age-sex interaction (*p* = 0.0523). For *IL10* (*n* = 179, due to missing data for nine subjects), Fig. 3b indicates methylation generally decreases with age (*p* < 0.0001) but is not associated with sex.

For the rest of the analysis, we used the residuals from fitting methylation to a regression model with the following covariates: sex, age, BMI, and age-sex interaction. For much of the analysis, we used averages across multiple sites, so these residuals are divided by the estimated standard deviation of the error to obtain “normalized” methylation, giving equal weight to all sites in these averages.

### Association between methylation and asthma status

We tested whether asthma was significantly associated with normalized methylation at each CpG site studied for *Foxp3* (Fig. 3a, Additional file 1: Table S4) using an ANOVA model. We found that asthma was significantly associated with methylation at four of the six CpG sites in the promoter region of *Foxp3* (*p* < 0.05). We also found that averaging over all six of these sites gave a stronger association than that for any individual site, so we used this average in the rest of the analysis. To aid in interpretation of the effect sizes in Fig. 3a, we note that the estimated standard deviation of the error for each sex at each CpG site in the *Foxp3* promoter was generally 4–6%. Hence, an effect size of 0.3 for the normalized values is roughly equivalent to 1.2–1.8 for percent methylation.

Similarly, we found that asthma was significantly associated with normalized methylation at all three CpG sites in region 3 of intron 4 of *IL10* (*p* < 0.05) (Fig. 3b, Additional file 1: Table S4). Furthermore, averaging over all three of these sites gave a stronger association than for any individual site, so we used this average in the rest of the analysis. The estimated standard deviation of the error for each sex at each CpG site in *IL10* was generally 9–11%.

Because the measured PBMC methylation is the average of all constituent cell types in a sample weighted by their proportion, an observed difference in methylation between healthy and asthmatic subjects could potentially be an artifact of differences in the underlying cell distribution. Conversely, because Treg cells constitute a small percentage of the PBMC fraction, a large difference in methylation within Treg could be masked by a small difference in cell composition. To rule out these possibilities, we performed two kinds of analyses on a random subset of the data for which cell counts of nine different cell types were obtained using CyTOF. First, we compared the proportion of each cell type. The proportion of activated Tregs was found to be statistically indistinguishable between asthmatic and healthy subjects (*p* = 0.528). After multiple testing corrections at a false discovery rate (FDR) of 0.05, Th17 cells were the only type of cell among those tested with a statistically significant difference (see Additional file 1: Table S2 for adjusted *p* values). We then repeated multiple linear regression to fit the average methylation in the promoter area of the *Foxp3* gene and the average methylation of region 3 of intron 4 of the *IL10* gene. The cell type composition was included as an additional covariate to adjust for any differences. For *IL10*, there was a higher DMR of *IL10* by 8.9±7.7% (*p* = 0.026) for asthmatic subjects.

### Association between methylation and ambient air pollution

We investigated the association between AAP exposure (CO, O_3_, NO_2_, PM_2.5_) and methylation of CpG sites in the *Foxp3* and *IL10* genes. The general characteristics of our estimates of AAP exposure were described previously in this “Results” section.

**Fig. 4:**
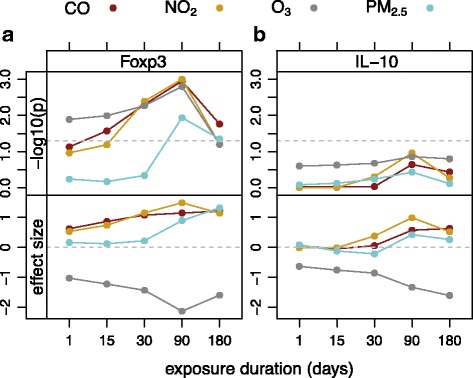
The *p* values and effect sizes of the associations between each pollutant and normalized methylation, averaged over the sites in the promoter of Foxp3, and over the sites in region 3 of intron 4 of IL-10. Each model also includes an effect for season and one for asthma. The horizontal dashed lines in the *p* value plots indicate *p* = 0.05. In the plots of effect sizes, the dashed lines indicate *y* = 0. To compare effect sizes across pollutants, each AAP value is divided by the 90th percentile of all sensor measurements for the pollutant

To test for association with methylation, we included one pollutant at one time period along with asthma in the linear regression model. To control for other seasonal effects such as pollen (for which we do not have a direct measurement), we also considered including season in the model, which greatly increased *p* values for pollutants. (Additional file 1: Figures S8 and S9 show *p* values for all models, all pollutants, and all time periods considered.) Adding a random family effect had little effect on *p* values for pollutants. Therefore, we focused on the results from a model that included asthma, season, and one pollutant. The *p* values and effect sizes of the pollutant on averaged normalized methylation are plotted in Fig. 4. For both genes and all pollutants, the smallest *p* value for pollutant occurred at the 90-day exposure duration, prior to the blood draw. For *IL10*, this association was not significant for any of the four AAPs (*p* > 0.1), but all were significant (*p* < 0.02) for *Foxp3*. Table 2 shows the *p* values and regression coefficients for the 90-day pollution exposure prior to the blood draw and *Foxp3* promoter methylation models. The *p* values for the season are not significant in this model, and methylation is clearly more strongly associated with 90-day exposure. To compare the effect sizes to each other, we divided each AAP value by the 90th percentile of all monitor measurements for the pollutant, which limits the interpretability of any individual pollutant coefficient. Finally, we also considered aggregating the pollutants to increase power. We used only the first principal component (PC1) of the AAP values scaled so that each pollutant has mean zero and variance one. PC1 accounts for 93% of their total variance; the sign of the weight of O_3_ was the opposite of the sign of the other three weights, and the absolute values of the weights are within 4% of each other. The last row of Table 2 shows the *p* values and regression coefficients for a model including PC1 along with asthma and season. The *p* value for PC1 is roughly the minimum of the pollutant *p* values in the other rows.

**Table 2:** *p* values and regression coefficients for 90-day pollution exposure prior to blood draw and *Foxp3* promoter methylation models

	Asthma		Pollutant		Season				
Pollutant in model	*p*	Coef	*p*	Coef	*p*	Spring	Summer	Autumn	Winter
CO	0.008	0.283	0.001	1.157	0.400	− 0.828	− 0.863	− 0.858	− 1.140
NO_2_	0.010	0.276	0.001	1.504	0.287	− 0.957	− 0.852	− 1.052	− 1.277
O_3_	0.011	0.275	0.002	− 2.158	0.437	1.045	1.436	1.444	0.829
PM_2.5_	0.016	0.261	0.012	0.903	0.145	− 0.445	− 0.608	− 0.665	− 0.770
PC1	0.011	0.273	0.001	NA	0.331	− 0.075	0.034	− 0.051	− 0.442

The *p* values and regression coefficients from models analyzing the associations between 90-day average pollutant exposure and normalized methylation averaged over the sites in the promoter of *Foxp3*. Each row is for one model that includes 90-day exposure for the specified pollutant. Asthma and season are covariates in every model. Since season is modeled as a factor with four levels, it has a coefficient for each season. In the last row, the pollutants are aggregated by their first principal component. PC1 is scaled much differently than the exposure values used in the other rows and its sign is arbitrary, so we omit its coefficient.

### Differences in percent methylation across repeated testing of methylation and estimation of ambient air pollution

In a convenience subset of the subjects, we evaluated changes in DMRs on the *Foxp3* (*n* = 33) and *IL10* (*n* = 30) genes after a period of time (mean 820 days, SD 243 days). We evaluated whether our model could explain changes in normalized methylation associated with exposure to AAPs. We predicted percent methylation for each participant at the time of repeat measure using data from 188 subjects (or 179 for *IL10*) and average normalized methylation to covariates asthma, season, and 90-day average of exposure to a single pollutant. Additional file 1: Figure S5 compares the observed values from the second time point to both the predicted values and to the original values for each gene and each pollutant for the subset of subjects with two visits. For any given individual, the actual normalized values generally change between the two time points. Furthermore, the prediction error for the second time point is generally large, but the model does correctly predict for most individuals whether an individual will have higher or lower DMRs at the second time point than at the first sample time.

### Treg: *Foxp3* and *IL10* protein expression

Cell type and protein expression were measured by CyTOF. The association between both activated (a result of the PMA-ION used to stimulate *IL10* cytokine production) Tregs (CD25 high, CD127 low) in CD4+ cells and *Foxp3* protein expression vs. *Foxp3* methylation was analyzed at each CpG site for the 74 subjects for which we had performed CyTOF analysis. When analyzed using multivariate linear regression with the covariates of asthma status, age, BMI, and sex, Tregs were inversely proportional to the average *Foxp3* methylation of the CpG sites in the promoter site (*p* = .039; Additional file 1: Figure S6a). There was no significant association between *Foxp3* methylation and *Foxp3* expression (Additional file 1: Figure S6b).

The association between *IL10* cytokine expression in CD4+ T cells and average *IL10* methylation at region 3 of intron 4 was also analyzed for a subset of subjects with available cytokine data (*n* = 53). When analyzed using multivariate linear regression with the covariates of asthma status, age, BMI, and sex, *IL10* cytokine expression was positively associated with *IL10* methylation (*p* = 0.030; Additional file 1: Figure S6c).

## Discussion

In this cohort study of children from Fresno, an area located in the San Joaquin Valley of California and known for high AAP exposure, we examined DMRs of *Foxp3* and *IL10*, which are genes involved in immune tolerance [28, 29]. *Foxp3* plays a key role in maintaining tolerance to common antigens in asthma and allergy [30]. *IL10* secretion by T cells can promote tolerance and suppress the production of IgE by B cells [29]. Additionally, prior studies have found an association between exposure to AAPs and *Foxp3* or *IL10* modifications [14, 31]. Overall, the results were consistent with our primary hypothesis (i.e., higher DMR with increased pollution exposure).

Our results indicate that DMRs in the *Foxp3* promoter and *IL10* intronic regions are preferentially methylated in asthmatic patients. These results are supported by other studies that have similarly found DNA methylation differences between asthmatic and control subjects at CpG loci in other genes [10, 32]. However, this DMR may be associated with asthma, regardless of the time point of pollution exposure. Furthermore, recent work indicates that heritable epigenetic alterations may mediate the increasing prevalence of atopic diseases [33, 34]. These epigenetic markers may serve as potential biomarkers to support clinical diagnosis.

Several variables measured in this study have been shown to be associated with asthma, such as age, BMI, and sex. We therefore adjusted for these factors in our analyses and found that exposure to AAPs is associated with increased DMRs in the promoter region of *Foxp3*. These results are consistent with prior studies that have demonstrated that the *Foxp3* gene is involved in overall immune tolerance [28], and more specifically that Tregs, which are suppressors of immune responses involved in asthma pathogenesis, are impaired in pediatric asthma [35]. *Foxp3* can also be present in activated CD4+ T effector cells; therefore, its presence is not a sine qua non of a Treg. We have also shown in two prior studies that increased exposure to ambient PAHs is associated with generalized higher differential DMR of the *Foxp3* locus [8, 14].

This study provides novel information by identifying specific gene regions that are sensitive to AAPs. We evaluated methylation at the enhancer and promoter regions of the *Foxp3* gene. We found methylation at the upstream promoter region of *Foxp3* to be associated with both asthma status and exposure to AAPs. However, we found no correlation between methylation and asthma status at the enhancer region. Importantly, our findings of a positive association between AAP exposure and *Foxp3* methylation in our initial cross-sectional analyses were consistent with the results of our longitudinal analyses in a subset of subjects.

We also found that individual pollutants differ in their effects on CpG methylation. Exposure to higher levels of NO_2_, CO, and PM_2.5_ are associated with higher DMRs of the promoter region of the *Foxp3* gene. However, O_3_ exposure was inversely associated with *Foxp3* methylation. This is not surprising as O_3_ is a regional pollutant with a seasonal pattern opposite to that of the other pollutants (Additional file 1: Figure S7), and we may not be observing the effect of high ozone on methylation, but, rather, the fact that the other pollutants were low.

This is the first study to our knowledge that has evaluated the association between *IL10* methylation and air pollution. We found that DMRs in the intronic region 3 are preferentially methylated in patients with asthma. However, we did not find significant associations between AAP and intron 4 methylation. In future studies, additional CpG sites, such as those in the promoter region of *IL10*, could be evaluated.

While previous investigations have suggested that exposure to higher levels of AAPs are associated with increased prevalence of asthma and allergy [36–39] and that protein expression of *IL10* decreased as PAH exposure increased from 24 h to 1 year [14], previous studies have not specified a critical time of exposure duration. Here, we determined that there was a significant positive association of *Foxp3 promoter* methylation and pollution for all subjects when there was exposure to certain AAPs (NO_2_, CO, and PM_2.5_) over the previous *90 days prior to the blood draw*. In addition, AAP exposure averaged over the 90 days was more strongly associated with *Foxp3* methylation than it was with season. For the *IL10* gene, there was not a significant association between pollutant exposure levels and the amount of intro region methylation, prompting our suggestion that additional *IL10 gene regions* be evaluated in future studies.

By using CyToF, we were able to link methylation and protein levels in high dimensional cell immune monitoring for these subjects. In addition, we demonstrated that methylation of the promoter region of the *Foxp3* gene is negatively associated with activated Treg levels, and *IL10* methylation at region 3 of intron 4 is positively associated with *IL10* cytokine expression. Foxp3 is produced by both Treg and activated T cells. However, *IL10* is synthesized by many immune cells and given there was a wide standard of error, it is difficult to comment on the biological significance of the *IL10* results.

We also recognize that our study is subject to a number of limitations. Blood collection occurred across all four seasons, and visits were not evenly distributed within each season. It is possible that the visit peak in April is driven by seasonal variations in airborne pollen and that the regression analyses might not remove the impact of this effect. The 90-day exposure finding could represent opposing effects (pollen/pollutant seasonal variations), which negate each other so that pollutant exposure impact can be detected. Future analyses are planned to address this. Not only do the AAP pollutants vary by season, aeroallergens such as ragweed, oak/maple, and Pinaceae pollen can affect the severity of asthma and pediatric asthma emergency department and clinic visits and have been shown to be associated with daily aeroallergen concentrations [40]. Repeat visits are limited in number and did not necessarily occur in the same season. To compensate, seasonality was included in the models used to investigate associations between methylation and AAP exposure, and 90-day exposure prior to the blood draw had a stronger effect than season, but simply using four seasons to model seasonality might not accurately reflect actual pollen levels in a given year. Furthermore, individual exposure was evaluated with the inverse distance weighting (IDW) method, rather than directly measured [25, 26]. It is also possible that other pollutants, dietary exposure, and toxins from the air, water, and other sources, not measured in this study, may contribute to DNA methylation. The generalizability of the results may be limited because the population was predominantly Hispanic and a convenience sample. And ethnicity was not adjusted in the models. Asthma was defined by self-report of physician diagnosis of asthma, rather than by clinical testing. However, in publications using the same technique, it was found that the agreement of patient self-report and physician diagnosis was correlated [41] with the questions about “physician-diagnosed asthma” having a specificity of 99% [42]; therefore, we used this method as part of our study definition of asthma. Our failure to find a significant association between *Foxp3* protein expression and *Foxp3* methylation may be related to the ongoing debate whether all Treg are *Foxp3* positive or whether all *Foxp3* positive T cells are Treg or if transcription and protein expression are directly related in Treg [43]. Given that the Treg population is a small fraction of the overall cell count, the measurement of *Foxp3* protein expression may be lost in the margin of error when measuring both it and the percentage of Tregs.

In summary, our data demonstrate that (1) CpG methylation in the promoter region of *Foxp3* and in the intronic region of *IL10* is greater in asthmatic than non-asthmatic subjects; (2) methylation levels at specific CpG sites in the *Foxp3* promoter region are associated with level of exposure to the pollutants CO, NO_2_, PM_2.5_, and O_3_ over the previous 90 days prior to the blood draw; and (3) activated Treg levels are negatively associated with average *Foxp3* methylation in the promoter region, and *IL10* methylation at region 3 of intron 4 is positively associated with *IL10* cytokine expression. The findings from this study increase our understanding of the epigenetic effects of exposure to AAPs and may impact the development of preventative regimens and/or therapies to alter methylation of these sites and possibly prevent asthma associated with AAP exposure [44].

## Conclusion

Our study suggests that asthma is significantly associated with higher DMRs within the promoter region of *Foxp3* as well as region 3 of intron 4 of *IL10*. In addition, DMRs in the promoter region of *Foxp3* are positively linked with average exposure to CO, NO_2_, and PM_2.5_ during the 90 days prior to the blood draw. These findings may provide important insight into understanding how pollution could exert detrimental effects relating to asthma, but need to be confirmed in future studies. Ultimately, this research may be useful in elucidating epigenetic biomarkers of asthma and in developing therapeutic targets by which to prevent or correct epigenetic damage resulting from pollution.

## Additional files

Additional file 1: Online data supplement. (DOCX 2086 kb)

Additional file 2: Code R. (R 71 kb)

Additional file 3: Public data. (CSV 56 kb)

Additional file 4: AQbySensor. (CSV 1709 kb)

Additional file 5: Exposure. (CSV 120 kb)
