# Supplementary material for: Exposure to NO2, CO, and PM2.5 is linked to regional DNA methylation differences in asthma
Source: Clin Epigenetics. 2018 Jan 5;10:2. doi: 10.1186/s13148-017-0433-4 (PMC5756438; doi:10.1186/s13148-017-0433-4)
Supplement: Supplementary file 1 — Online data supplement. (DOCX 2086 kb) [file 13148_2017_433_MOESM1_ESM.docx]

Online Data Supplement

# TITLE: Exposure to NO_2_, CO, and PM_2.5_ Is Linked to Discrete Methylation Differences in Asthma

**Authors:** Mary Prunicki, Laurel Stell, Deendayal Dinakarpandian, Mariangels de Planell-Saguer, Richard W. Lucas, S. Katharine Hammond, John R. Balmes, Xiaoying Zhou, Tara Paglino, Chiara Sabatti, Rachel L. Miller, Kari C. Nadeau

**SUPPLEMENTARY METHODS**

**Collection and Processing of Blood Specimens**

- Whole blood specimens were shipped from Fresno, CA to our processing laboratory at Stanford University according to standardized procedures. Defrosted PBMCs yielded over 88% viability, and only live cells were used for experiments.

**Methylation Analysis**

- Genomic DNA from PBMCs was extracted using QIAamp DNA blood Mini Kit (Qiagen, Valencia, CA) and then subjected to bisulfite modification using an EZ DNA Methylation Kit (Zymo Research, Orange, CA), both according to the recommendations of the manufacturer and as described in published literature [1]. Subsequent DNA amplification of specific CpG regions with each of the 4 genes was conducted by PCR using the different pairs of primers for each region enumerated in Table S1 and Figure 1. Each PCR reaction included Qiagen Hot Star Taq DNA polymerase reagents (Qiagen Valencia, CA) with the following PCR mixture: 1 X PCR buffer, 0.5 μM deoxynucleotide triphosphates (dNTP), 0.5 μM each of forward and reverse primers, RNAse-free water and 20ng of bisulfite-converted template DNA. The methylation of the PCR products was assessed by pyrosequencing with the PyroMark CpG Assay in the Q96 MD system (Qiagen, Valencia, CA) using sequencing primers displayed in Table S1. All the primers were designed using PyroMark Assay Design software SW 2.0 (Qiagen, Orange, CA).
- **Mass Cytometry**
- Antibodies were purchased from DVS Biosciences (San Francisco, CA), from Longwood (Boston, MA), or conjugated in-house using purified proteins. Antibodies were titrated prior to use. Frozen human PBMCs were thawed from the same sample collection as that used for the methylation studies (different aliquot), and 2 million cells were stimulated with a freshly prepared solution of the following for 4 hours at 37 degrees C: 150 ng/ml of Phorbol 12-Myristate 13-Acetate (PMA; Cat No: P8139; Sigma-Aldrich, St. Louis, MO), 100 ng/ml of ionomycin (Cat No: I0634; Sigma-Aldrich, St. Louis, MO; Brefeldin A (Cat No: 4506–51; eBioscience, CA).

The cell staining procedure follows the protocols provided by DVS Science, including Cisplatin (Cat No: 201064; DVS Science, CA) for live/dead cell staining, surface biomarker staining, intracellular marker staining and DNA intercalator-Ir (Cat No: 201192; DVS Science, CA) staining. CyTOF settings followed the manufacturer’s protocol. FlowJo vX10.0 was used for data analysis. Singlets were gated using DNA intercalator Ir193 vs. cell length; then, cells negative to cisplatin were gated as live cells. Activated Tregs were defined as CD3+, CD4+, CD25high and 127 low cells.

**Ambient Air Pollution Exposure Estimation**

The air pollution exposure data consisted of hourly concentration values, from which we computed daily averages for each pollutant. Data were obtained from four Environmental Protection Agency (EPA) monitoring stations located within the Fresno city limits. Because the daily averages have very large variance, we smoothed them by taking centered, 7-day rolling averages. The exposure to one of the four pollutants at a given location on a given day was estimated by a weighted average of the daily rolling daily averages from all monitoring stations available for that day. The weight of each monitoring station was proportional to the inverse of the square of the distance between the location of the monitoring station and that of the subjects’ home residences at the time of sample collection [2]. To compute the inverse distance weighting (IDW), we used the R package “gstat” [3]. To illustrate the method, we chose a single day (January 1, 2014) and applied IDW to estimate exposure of each pollutant on a rectangular grid covering all of the home addresses. Figure S2 is a heat map of the resulting values.

We used IDW in order to have good temporal resolution and be able to investigate pollution exposure over different durations. IDW is a relatively simple spatial model, however, and cannot determine the differences in exposure at two places in close proximity, such as home and school addresses; therefore, we used only the subjects’ home locations. Emphasizing temporal over spatial resolution is also consistent with the fact that the variability in pollution over time is much greater than the variability between the air quality monitoring stations used in this study. The spatial resolution of IDW is also consistent with using only four monitoring stations for an area larger than 400 km^2^.

**Variables and Samples Used in Analyses**

There are 198 subjects that have at least one sample with sex, age, BMI, asthma status, percent methylation at all 10 *Foxp3* CpG sites, and a home address within 10 km of at least one of the air quality monitoring stations used in this study. A sample must also have a value for methylation at all six *IL10* CpG sites in order to be used in the analysis of *IL10* methylation.

As shown in Figure 3, some of these samples have unusual values of *Foxp3* percent methylation. If they are included in ordinary linear regression analysis, they invalidate the assumption of Gaussian distributions underlying the p-values. One way to handle outliers is to use robust nonparametric methods, but none have been developed that can also account for the family relatedness described below. Consequently, we will obtain more accurate p-values if we exclude these points, as we have done in the main text. We define an outlier to be a value farther than three times the interquartile range (IQR) from the first quartile or third quartile (more precisely, outside the whiskers computed by the R function “boxplot.stats,” with argument “coef” set to 3). The 48 values that meet this criterion are shown in Figure 3. They come from 18 individuals, but 8 of these individuals have another sample that lacks outliers, which leaves 188 subjects for analysis. In this supplement, we report the results we would have obtained if we had not excluded these outliers, showing that the main conclusions would not have changed substantially.

To avoid the correlations between samples from the same individual, we chose one sample per subject (except for the analysis of repeat measurements), selecting the earliest visit with the most complete data.

**Family Relatedness**

The 188 subjects include 42 groups (for a total of 97 individuals) that appear related, as determined by information on questionnaires. The precise degree of relatedness was unknown, however. To account for family relatedness, we used a random effects model, which adds a random intercept term for each family [4]. We used the function “gls” in the R package “nlme.” To be able to use the “anova” function in this package, we set the gls option “method” to “ML” throughout. Note that the inclusion of a family effect typically reduces the power to detect association with variables of interest, since family is confounded with genetic predisposition to asthma and with pollution exposure. Consequently, the main text reports results for the mixed model only when the family effect is clearly significant, but this supplement includes results when using random family effects for each analysis.

**Normalization of Methylation**

Before testing our primary hypotheses, we first adjust for any association between methylation and sex, age, or BMI. To identify an appropriate model for this adjustment, we performed linear regression of the percent methylation on several different models (N=188 for *Foxp3*; N=179 for *IL10*). We used “gls” (with option “method” set to “ML”) in the R package “nlme” since it allowed us to fit all sites simultaneously, while allowing the error variance to depend upon site and sex. We then used the “anova” function in this package to compare the models, obtaining the p-values shown in Table S3.

While the only strong association for *IL10* in these models is with age, *Foxp3* has a strong association with sex, age, and a sex-age interaction; and *Foxp3* may also have a weak association with BMI. Since the goal here is not generalization, but only to adjust methylation in order to reduce the number of covariates in subsequent analysis, overfitting is not a concern; in fact, it would tend to reduce power in subsequent analysis, rather than to lead to Type I errors. Hence, we use the same model for both genes for simplicity: the model with covariates sex, age, BMI, and a sex-age interaction. Similarly, the error variance clearly depends upon sex and site for *Foxp3* (*p* < 0.0001), but for *IL10* it does not depend upon either (*p* > 0.3); nonetheless, we use the same model for both. Again bearing the goal in mind, we do not compare results including outliers or using random family effects.

The normalized values are the residuals from the selected models, divided by the estimated standard deviation of the error. Plotting the empirical distributions of these residuals for males and females separately shows that they are essentially the same at each CpG site—at least those in the promoter of *Foxp3* and in region 3 of *IL10*, which are the locations of all of our discoveries. Furthermore, QQ plots show that these distributions are approximately Gaussian at these sites.

**Association Between Methylation and Asthma Status**

In addition to the analysis described in the main text, we also considered a mixed effects model fitting normalized methylation to a fixed asthma effect and a random family effect. The random family effect is significant (p<0.05) for all of the response variables for *IL10* in Table S4, and also for most of the response variables involving CpG sites in the *Foxp3* promoter. In particular, p = 0.0006 for the average of normalized methylation over the sites in the promoter region of *Foxp3*, and p = 0.0069 for the average of normalized methylation over the sites in region 3 of intron 4 of *IL10*. Table S4 shows that including a random family effect generally increases the p-value for asthma, which is likely due to the fact that asthma tends to run in families. Of the 133 “families”, 42 had more than one child in the study. In 30 of these 42, either all the children had asthma or else all the children did not have asthma. This means that the asthma effect and the family effect are somewhat interchangeable in these 30 families. Even with the family effect, p < 0.05 for the averages over the promoter region of *Foxp3* and over region 3 of intron 4 of *IL10*.

Table S4 also shows the p-values that would result if we had not excluded samples with *Foxp3* methylation outliers. While we emphasize that the p-values are not accurate when the errors are not Gaussian, we still obtain p < 0.05 for the averages over the promoter region of *Foxp3* and over region 3 of intron 4 of *IL10*, although the latter has p = 0.087 when a random family effect is used. Since there were no clearly unusual values for *IL10* methylation, it is initially surprising that the p-values associated with sites in its region 3 of intron 4 changed so much between the two data sets. The explanation is that 6 of the samples used now but excluded in the analysis in the main text are healthy controls that have normalized methylation averages in this region that are larger than most other healthy controls, though not extremely so.

**Association Between Methylation and Ambient Air Pollution**

Air quality is cyclic over the course of a year, as are other factors that could affect asthma and methylation, such as pollen. To control for the possible effect of these confounders in models with pollution as a covariate, we also included the season in which the sample was collected. Since the dates of equinoxes and solstices can change, we used the “meteorological seasons,” with spring starting on March 1, summer starting on June 1, and so forth.

Figure S8 and Figure S9 show the p-values for the following models for normalized methylation averaged over the promoter region of *Foxp3*, and averaged over region 3 of intron 4 of *IL10*:

**Selected** covariates were asthma, season and a pollutant

**Model A** covariates were asthma and a pollutant

**Model B** covariates were asthma and a pollutant with random family effect

**Model C** covariates were asthma, season and a pollutant with random family effect

Including season greatly increased the p-values for the pollutants, but they still had *p* < 0.05 for the 90-day exposure duration when *Foxp3* methylation was the response. In other words, 90-day pollution exposure explains methylation in the *Foxp3* promoter better than does season, but this is not the case for methylation in *IL10* region 3. Including random family effects did not change the p-values of other variables very much, even though the family effects had p < 0.05.

We also considered adding an interaction between asthma and pollutant to each fixed effects model with asthma, season, and 90-day exposure for a single pollutant. The interaction term was not significant (*p* > 0.2) for any pollutant or either gene.

Finally, we checked the effect of excluding *Foxp3* outliers. Figure S10 is the analogue of Figure 5. While there are obvious differences between them, they do not change the conclusions stated in the main text. The changes in the analogues of Figure S8 and Figure S9 have similar magnitude and no effect on the conclusions, so we do not include them here.

**Differences in Percent Methylation Across Repeated Testing of Methylation and Estimation of Ambient Air Pollution**

For the subjects with two samples (N=33 for *Foxp3*, N=30 for *IL10*), we analyzed the change in methylation over time using the models obtained previously by fitting one sample per individual. First, for sample *k* from participant *j* in the set of repeated tests, we computed the mean $y_{jk}$over the appropriate sites of the normalized methylation. If the within-subject variation in methylation were entirely random, then the best predictor of the change in $y_{jk}$for the same individual at a later time would be zero. This “baseline” predictor of the change gave root-mean square (RMS) error = 1.1 for *Foxp3*, and 1.2 for *IL10*. We have estimated previously the coefficients in a linear combination of asthma, 90-day pollution exposure, and season to model $y_{jk}$. This would predict that the change in $y_{jk}$ is equal to the resulting linear combination of the change between samples of pollution exposure and season, since asthma status is constant for each individual. The RMS error for the predicted change was then 0.88–0.92 for *Foxp3*, depending on the pollutant, and 1.0 for *IL10* and any pollutant. The relative decrease in the RMS was 19–22% for *Foxp3* and 12–13% for *IL10*. This was further evidence that 90-day pollution exposure or season or both help to explain variability in methylation at least at some CpG sites in these genes. Furthermore, the association was stronger for *Foxp3* than for *IL10*. Figure S5 compares $y_{j2}$ with $y_{j1}$ as well as the predicted value of $y_{j2.}$

**References**

1. Nadeau K, McDonald-Hyman C, Noth EM, Pratt B, Hammond SK, Balmes J, et al. Ambient air pollution impairs regulatory T-cell function in asthma. J Allergy Clin Immunol 2010;126:845-52.

2. Shepard D. A two-dimensional interpolation function for irregularly-spaced data. Proceedings of the 1968 23rd ACM national conference: ACM; 1968. p. 517-24.

3. Pebesma EJ. Multivariable geostatistics in S: the gstat package. Computers & Geosciences 2004;30:683-91.

4. Pinheiro JC, Bates DM. Mixed-Effects Models in S and S-PLUS. New York: Springer; 2000.

**Table S1:** Sequences of the primers used for PCR amplification and pyrosequencing

| **Primer Name and Type** | **Sequence** |
| --- | --- |
| *Foxp3* Upstream Enhancer  PCR Forward  PCR-Reverse  Pyrosequencing | 5’-[Biotin] -ATGGAGGTGGAGGTTGAAG-3'  5’ -ACTCTACTTTTTTCCCCATCT-3'  5' -CCCTAACTCCCTCAT-3' |
| *Foxp3* Promoter 1  PCR Forward  PCR-Reverse  Pyrosequencing | 5’-[Biotin] -TTTTTGTGGTGAGGGGAAGAAATTA-3'  5’-AACCGCAAACCTCTCTCTTCTAATAATCCA-3  5' -CCAAATTTTTTTCCATAAATATAT-3' |
| *Foxp3* Promoter 2  PCR Forward  PCR-Reverse  Pyrosequencing | 5’-AAATTTGGATTATTAGAAGAGAGAGG-3’  5’-[Biotin] -AACTAACAAAAAAAAATCAACCTAACTTAT-3  5’-AGAAGAGAGAGGTTTG-3’ |
| *Foxp3* Promoter 3  PCR Forward  PCR-Reverse  Pyrosequencing | 5’-AAATTTGGATTATTAGAAGAGAGAGG-3’  5’-[Biotin] -AACTAACAAAAAAAAATCAACCTAACTTAT-3’  5- -TTTTTAGGTATAAAAGTAAAGTTGT-3’ |
| *IL10* Int4 R1  PCR Forward  PCR-Reverse  Pyrosequencing | 5'-GTGGGAATTTGATAGATGGTATGATTT-3'  5'-[Biotin]-TTTCCTTTATCACTCTCCCCAACAT-3'  5'-TGGGGGTTTTTAAATGA-3' |
| *IL10* Int4 R3  PCR Forward  PCR-Reverse  Pyrosequencing | 5'-GGAGGTGTTTGAGAATGTTAGT-3'  5'-[Biotin]-CTATATTCTCCCACCAACACAC-3'  5'-GTGAGTGAATTTTTTTAGTTTGAT-3' |

**Table S2:** Comparison of cell type composition between asthmatic and non-asthmatics groups.

| Cell Type | Benjamini,-Hochberg Adjusted P-value |
| --- | --- |
| Th17(CD3+CD4+CD8-CCR6+cd161+cxcr3-ccr4+cd25-) | 0.012 |
| Th2(CD3+CD4+CD8-ccr4+ ccr5-cxcr3- cd25-ccr6-) | 0.085 |
| Th1 (CD3+CD4+CD8-ccr4- ccr5+cxcr3+cd25-) | 0.125 |
| CD3+CD4+ (non Th1/Th2/Th17/Treg) | 0.125 |
| CD3+ (non CD4/CD8) | 0.149 |
| CD14+ monocytes | 0.575 |
| CD20+ B cells | 0.661 |
| Treg(CD3+CD4+CD8-CD25high*Foxp3*+CD127low) | 0.661 |
| CD8+ (CD4-) cells | 0.809 |

**Table S3**: Model comparison of the associations between each covariate and normalized methylation for the *Foxp3* (n=188) and *IL10* (n=179) genes.

|  | **p-value** | |
| --- | --- | --- |
| Covariates | *Foxp3* | *IL10* |
| Intercept Only |  |  |
| Sex | <.0001 | 0.4201 |
| Sex, Age | <.0001 | <.0001 |
| Sex, Age; Sex-Age Interaction | <.0001 | 0.0422 |
| Sex, age, BMI; Sex-Age Interaction | 0.0523 | 0.9808 |
| Sex, Age, BMI; Sex-Age, Sex-BMI Interactions | 0.4799 | 0.0180 |

Each model uses all the CpG sites measured in the specified gene, with different coefficients—including an intercept—for each CpG site; the error variances depend on site and sex. Each formula is compared to that above. The next to last model is used for normalization.

**Table S4:** p-values from ANOVA tests of significance, with and without random family effects, of asthma status (the only fixed effect) on normalized methylation at individual sites, and also averages over groups of sites.

| Site | Without outliers | | With outliers | |
| --- | --- | --- | --- | --- |
|  | Fixed effect only | With random family effects | Fixed effect only | With random family effects |
|  |  | - - *Foxp3* |  |  |
|  | N=188 | | N=198 | |
| CpG -4506 | 0.564 | 0.681 | 0.679 | 0.772 |
| CpG -4500 | 0.508 | 0.497 | 0.520 | 0.574 |
| CpG -4494 | 0.400 | 0.354 | 0.733 | 0.580 |
| CpG -4484 | 0.422 | 0.425 | 0.707 | 0.737 |
| CpG -138 | 0.461 | 0.499 | 0.708 | 0.317 |
| CpG -126 | 0.042 | 0.029 | 0.082 | 0.047 |
| CpG -77 | 0.019 | 0.039 | 0.111 | 0.146 |
| CpG -65 | 0.059 | 0.030 | 0.116 | 0.095 |
| CpG -58 | 0.017 | 0.021 | 0.196 | 0.206 |
| CpG -15 | 0.009 | 0.039 | 0.015 | 0.030 |
| Promoter | 0.007 | 0.013 | 0.025 | 0.035 |
| All | 0.029 | 0.039 | 0.130 | 0.178 |
|  |  | *IL10* |  |  |
|  | N=179 | | N=189 | |
| CpG +2888 | 0.209 | 0.253 | 0.250 | 0.300 |
| CpG +2907 | 0.165 | 0.221 | 0.221 | 0.282 |
| CpG +2921 | 0.244 | 0.331 | 0.373 | 0.468 |
| CpG +3261 | 0.009 | 0.028 | 0.038 | 0.087 |
| CpG +3265 | 0.008 | 0.012 | 0.042 | 0.058 |
| CpG +3281 | 0.016 | 0.032 | 0.115 | 0.180 |
| Region 3 | 0.007 | 0.017 | 0.048 | 0.087 |
| All | 0.036 | 0.065 | 0.103 | 0.166 |

**FIGURE LEGENDS**

**Figure S1:** Comparison of annual pollution exposure between years and monitoring stations. Each group of bars is for one station (merging together the two in essentially the same location); each bar is the average for that station for the year indicated by the legend. The y-axis upper limit in each panel is the 90^th^ percentile of all daily values for that pollutant. Only two monitoring stations measured PM_2.5_.

**Figure S2:** Result of inverse, distance-weighted (IDW) averaging of the pollutants on January 1, 2014 over a rectangular grid covering all participant locations. Small dots show locations of the subjects’ homes at the time of the samples. Large, open circles show the locations of the EPA monitoring stations used in the IDW averaging for that pollutant on that date.

**Figure S3:** Distributions of pollution exposure across individuals for six different durations prior to the sample. Each black dot indicates the median. The box length shows the IQR, and the whiskers extend to the farthest data point that is no farther than 1.5 times the IQR from the box.

**Figure S4:** Relation between male methylation levels and female methylation levels for *Foxp3* sites. For each point, the x value is the average over all males (N=94) of the percent methylation at one CpG site, and the y value is the average over all females (N=94). The line is the best fit y = 38.46 + 0.49x, which is consistent with the active X chromosome in females having the same percent methylation as in males while the inactive X is 77% methylated.

**Figure S5:** Normalized methylation averaged over the sites in the promoter region of *Foxp3* and region 3 of intron 4 of *IL10*, across repeated testing. In each panel, the x-axis reports the value of average normalized methylation for one individual at the first test, and the y-axis reports the value at a later test. The dashed line corresponds to the position where points would be in case of equality. The empty black circles indicate realized values. The pink triangles indicate the expected values for the second test conditional on the first sample and pollutant information. Note that the pink triangles are closer to the 45-degree line, but generally we are able to predict the direction of change.

**Figure S6:** Plots of CyTOF results versus individual CpG sites in the promoter region of the *Foxp3* gene. A) Percent Treg cells in CD4+ cells versus *Foxp3* methylation, B) Percent *Foxp3* protein expression in Treg cells versus *Foxp3* methylation and C) Percent *IL10* cytokine in CD4+ cells versus *IL10* methylation.

**Figure S7:** Scatterplot of the values of 90-day exposure levels to each of the four different pollutants for each individual. The text in the boxes on the diagonal state the x-axis label for each panel in that column as well as the y-axis label for each panel in that row. The text in the upper panels gives the Pearson correlation between the pollutants in that row and column, with the size of the text proportional to the absolute value of the correlation.

**Figure S8:** Comparison of p-values from models (described in Supplement) of normalized methylation averaged over the sites in the promoter region of *Foxp3*. Each column corresponds to a model, identified by the included exposure duration reported on the x-axis. Different colors are used to indicate which pollutant is included in the models. Each row indicates a fixed or random effect in the model. If a panel is blank, then that covariate was not included in that model. Comparing columns to each other shows the effect of changing the model. The gray, dashed lines indicate p = 0.05. The magenta, dashed lines in the row for asthma indicate the p-value for the ANOVA test of asthma only.

**Figure S9:** Comparison of p-values from models (described in Supplement) of normalized methylation averaged over the sites in region 3 of intron 4 of *IL10*. Each column corresponds to a model, identified by the included exposure duration reported on the x-axis. Different colors are used to indicate which pollutant is included in the models. Each row indicates a fixed or random effect in the model. If a panel is blank, then that covariate was not included in that model. Comparing columns to each other shows the effect of changing the model. The gray, dashed lines indicate p = 0.05. The magenta, dashed lines in the row for asthma indicate the p-value for the ANOVA test of asthma only.

**Figure S10:** The p-values and effect sizes of the association between each pollutant and normalized methylation averaged over the sites in the promoter region of *Foxp3* and over the sites in region 3 of intron 4 of *IL10* *when Foxp3 methylation outliers are not discarded*. Each model also includes an effect for season and one for asthma. The horizontal dashed lines in the p-value plots indicate p = 0.05. In the plots of effect sizes, the dashed lines indicate y = 0.

**Figure S1**

**Figure S2**

**Figure S3**

**Figure S4**

**Figure S5**

**Figure S6**

**A)**

**B)**

**C)**

**
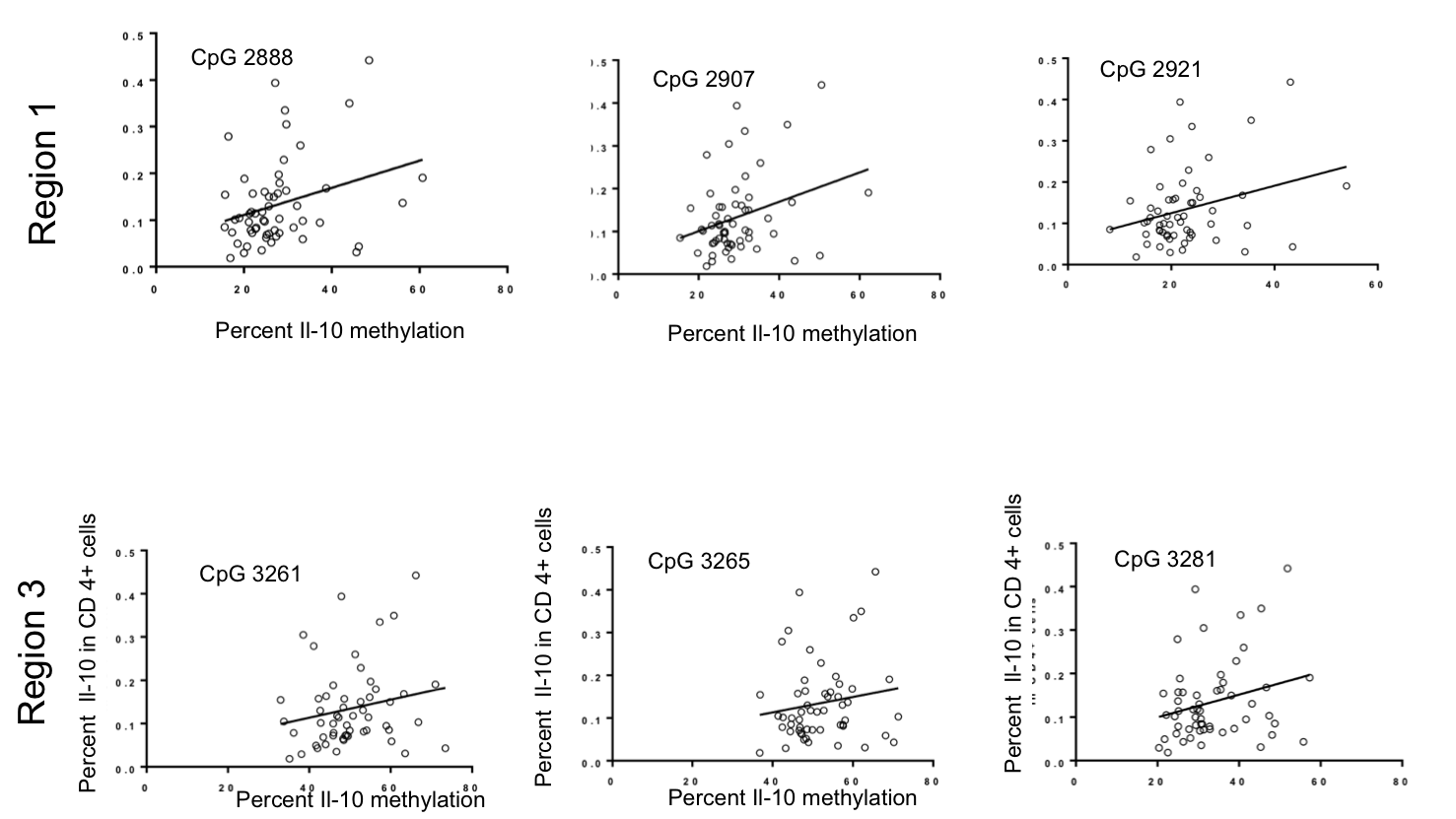
**

**Figure S7**

**Figure S8**

**Figure S9**

**Figure S10**
